# 4-Meth­oxy­quinolinium-2-carboxyl­ate dihydrate

**DOI:** 10.1107/S1600536811001541

**Published:** 2011-01-22

**Authors:** Madhukar Hemamalini, Hoong-Kun Fun

**Affiliations:** a X-ray Crystallography Unit, School of Physics, Universiti Sains Malaysia, 11800 USM, Penang, Malaysia

## Abstract

The title hydrated quinoline derivative, C_11_H_9_NO_3_·2H_2_O, crystallizes as a zwitterion in which the quinoline N atom is protonated. The quinoline ring is essentially planar, with a maximum deviation of 0.017 (2) Å. An intra­molecular N—H⋯O hydrogen bond between the protonated N atom and the O atom of the carboxyl­ate group in the zwitterion forms an *S*(5) ring motif. In the crystal, the zwitterions are connected into inversion dimers *via* pairs of N—H⋯O and C—H⋯O hydrogen bonds with *R*_2_^2^(4) and *R* ^1^_2_(6) motifs. The water mol­ecules are connected *via* O—H⋯O hydrogen bonds, forming supra­molecular chains along the *c* axis. Furthermore, the chains and the dimers are connected *via* O—H⋯O hydrogen bonds, forming ladder-like supra­molecular ribbons along the *c* axis.

## Related literature

For background to and the biological activity of quinoline derivatives, see: Morimoto *et al.* (1991 ▶); Michael (1997 ▶); Markees *et al.* (1970 ▶); Campbell *et al.* (1988 ▶); Zhou *et al.* (1989 ▶); Elman *et al.* (1985 ▶); Loh *et al.* (2010**a* ▶,b* ▶); Sasaki *et al.* (1998 ▶); Reux *et al.* (2009 ▶). For hydrogen-bond motifs, see: Bernstein *et al.* (1995 ▶). For the stability of the temperature controller used in the data collection, see: Cosier & Glazer (1986 ▶). 
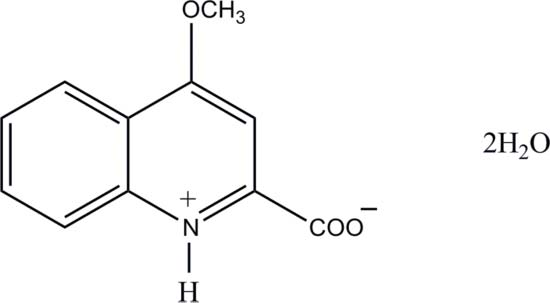


## Experimental

### 

#### Crystal data

C_11_H_9_NO_3_·2H_2_O
*M* *_r_* = 239.22
Monoclinic, 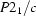

*a* = 5.7674 (11) Å
*b* = 21.196 (4) Å
*c* = 10.0993 (15) Å
β = 115.978 (8)°
*V* = 1109.9 (3) Å^3^
*Z* = 4
Mo *K*α radiation
μ = 0.11 mm^−1^
*T* = 100 K
0.23 × 0.13 × 0.09 mm

#### Data collection

Bruker APEXII DUO CCD area-detector diffractometer
Absorption correction: multi-scan (*SADABS*; Bruker, 2009 ▶) *T*_min_ = 0.974, *T*_max_ = 0.990
8743 measured reflections
3176 independent reflections
2123 reflections with *I* > 2σ(*I*)
*R*_int_ = 0.058

#### Refinement

*R*[*F* ^2^ > 2σ(*F* ^2^)] = 0.051
*wR*(*F* ^2^) = 0.140
*S* = 1.01
3176 reflections
155 parameters
H-atom parameters constrained
Δρ_max_ = 0.32 e Å^−3^
Δρ_min_ = −0.34 e Å^−3^

### 

Data collection: *APEX2* (Bruker, 2009 ▶); cell refinement: *SAINT* (Bruker, 2009 ▶); data reduction: *SAINT*; program(s) used to solve structure: *SHELXTL* (Sheldrick, 2008 ▶); program(s) used to refine structure: *SHELXTL*; molecular graphics: *SHELXTL*; software used to prepare material for publication: *SHELXTL* and *PLATON* (Spek, 2009 ▶).

## Supplementary Material

Crystal structure: contains datablocks global, I. DOI: 10.1107/S1600536811001541/is2654sup1.cif

Structure factors: contains datablocks I. DOI: 10.1107/S1600536811001541/is2654Isup2.hkl

Additional supplementary materials:  crystallographic information; 3D view; checkCIF report

## Figures and Tables

**Table 1:** Hydrogen-bond geometry (Å, °)

*D*—H⋯*A*	*D*—H	H⋯*A*	*D*⋯*A*	*D*—H⋯*A*
N1—H1⋯O1^i^	0.94	1.84	2.7608 (18)	164
O1*W*—H2⋯O2*W*	0.86	1.89	2.7478 (19)	176
O1*W*—H3⋯O2^ii^	0.91	1.86	2.7685 (16)	177
O2*W*—H4⋯O2^iii^	0.88	1.88	2.7498 (18)	171
O2*W*—H5⋯O1*W*^iv^	0.87	1.91	2.7860 (19)	176
C6—H6*A*⋯O1*W*^v^	0.93	2.59	3.418 (2)	149
C8—H8*A*⋯O1^i^	0.93	2.53	3.229 (2)	132
C11—H11*A*⋯O1*W*^vi^	0.96	2.58	3.317 (2)	134
C11—H11*B*⋯O2^iii^	0.96	2.53	3.272 (2)	134

Symmetry codes: (i) 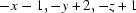; (ii) 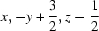; (iii) 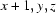; (iv) 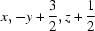; (v) 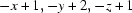; (vi) 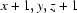.

**Fig. 1.**
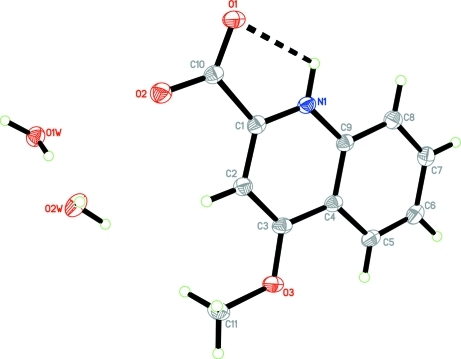
The asymmetric unit of the title compound. Displacement ellipsoids are drawn at the 50% probability level. Intramolecular hydrogen bonds shown by dotted lines.

**Fig. 2.**
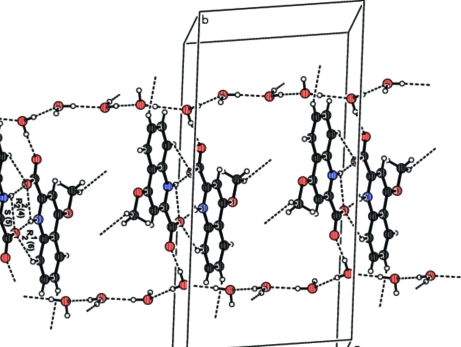
The crystal packing of the title compound, showing a hydrogen-bonded (dashed lines) ladder-like network.

## supplementary crystallographic information

### Comment

Quinolines and their derivatives are very important compounds because of their wide occurrence in natural products (Morimoto *et al.*, 1991; Michael, 1997) and biologically active compounds (Markees *et al.*, 1970; Campbell *et al.*, 1988). Quinoline-2-carboxylic acid (quinaldic acid) and tryptophan metabolite (Zhou *et al.*, 1989) are well-known chelating ligands (Elman *et al.*, 1985).  Recently, hydrogen-bonding patterns involving quinoline and its derivatives with organic acid have been investigated (Loh *et al.*, 2010**a*,b*). Syntheses of the quinoline derivatives have been discussed (Sasaki *et al.*, 1998; Reux *et al.*, 2009).

The title molecule, (Fig. 1), crystallizes as a zwitterion in which the quinoline N atom is protonated.  The asymmetric unit consists of one 4-methoxyquinolinium-2-carboxylate molecule and two water molecules. The quinoline ring (N1/C1–C9) is essentially planar, with a maximum deviation of 0.017 (2) Å for atom C4.

In the crystal structure (Fig. 2), the 4-methoxyquinolinium-2- carboxylate molecules are connected via N—H···O and C—H···O hydrogen bonds to form  *R*_2_^2^(4) and *R*^1^_2_(6) (Bernstein *et al.*, 1995) motifs. There is an intramolecular N—H···O hydrogen bond observed between the protonated nitrogen atom of the cationic part of the quinolinium and the oxygen atom of anionic part of the carboxylate group in the zwitterion forming an *S*(5) ring motif. The water molecules are connected *via* O—H···O hydrogen bonds to form one-dimensional supramolecular chains along the *c*-axis. Furthermore, the chains formed by water molecules and the 4-methoxyquinolinium-2-carboxylate molecules are connected *via* O—H···O (Table 1) hydrogen bonds to form ladder-like supramolecular ribbons along the *c*-axis.

### Experimental

A methanol solution (20 ml) of 4-methoxyquinoline-2-carboxylic acid (50. 8 mg, Aldrich) was warmed over a heating magnetic stirrer for 5 minutes. The resulting solution was allowed to cool slowly at room temperature. Crystals of the title compound appeared from the mother liquor after a few days.

### Refinement

All the H atoms were positioned geometrically (N—H = 0.9437 Å; C—H = 0.93 or 0.96 Å and O—H = 0.8586–0.9083 Å) and were refined using a riding model, with *U*_iso_(H) = 1.2 or 1.5*U*_eq_(C,O).

### Crystal data

**Table:**

C_11_H_9_NO_3_·2H_2_O	*F*(000) = 504
*M**_r_* = 239.22	*D*_x_ = 1.432 Mg m^−^^3^
Monoclinic, *P*2_1_/*c*	Mo *K*α radiation, λ = 0.71073 Å
Hall symbol: -P 2ybc	Cell parameters from 1896 reflections
*a* = 5.7674 (11) Å	θ = 3.0–29.6°
*b* = 21.196 (4) Å	µ = 0.11 mm^−^^1^
*c* = 10.0993 (15) Å	*T* = 100 K
β = 115.978 (8)°	Block, colourless
*V* = 1109.9 (3) Å^3^	0.23 × 0.13 × 0.09 mm
*Z* = 4	

### Data collection

**Table:**

Bruker APEXII DUO CCD area-detector diffractometer	3176 independent reflections
Radiation source: fine-focus sealed tube	2123 reflections with *I* > 2σ(*I*)
graphite	*R*_int_ = 0.058
φ and ω scans	θ_max_ = 30.0°, θ_min_ = 3.0°
Absorption correction: multi-scan (*SADABS*; Bruker, 2009)	*h* = −7→8
*T*_min_ = 0.974, *T*_max_ = 0.990	*k* = −29→29
8743 measured reflections	*l* = −10→14

### Refinement

**Table:**

Refinement on *F*^2^	Primary atom site location: structure-invariant direct methods
Least-squares matrix: full	Secondary atom site location: difference Fourier map
*R*[*F*^2^ > 2σ(*F*^2^)] = 0.051	Hydrogen site location: inferred from neighbouring sites
*wR*(*F*^2^) = 0.140	H-atom parameters constrained
*S* = 1.01	*w* = 1/[σ^2^(*F*_o_^2^) + (0.0701*P*)^2^] where *P* = (*F*_o_^2^ + 2*F*_c_^2^)/3
3176 reflections	(Δ/σ)_max_ = 0.001
155 parameters	Δρ_max_ = 0.32 e Å^−^^3^
0 restraints	Δρ_min_ = −0.34 e Å^−^^3^

### Special details

**Table:**

Experimental. The crystal was placed in the cold stream of an Oxford Cryosystems Cobra open-flow nitrogen cryostat (Cosier & Glazer, 1986) operating at 100.0 (1) K.
Geometry. All s.u.'s (except the s.u. in the dihedral angle between two l.s. planes) are estimated using the full covariance matrix. The cell s.u.'s are taken into account individually in the estimation of s.u.'s in distances, angles and torsion angles; correlations between s.u.'s in cell parameters are only used when they are defined by crystal symmetry. An approximate (isotropic) treatment of cell s.u.'s is used for estimating s.u.'s involving l.s. planes.
Refinement. Refinement of F^2^ against ALL reflections. The weighted R-factor wR and goodness of fit S are based on F^2^, conventional R-factors R are based on F, with F set to zero for negative F^2^. The threshold expression of F^2^ > 2σ(F^2^) is used only for calculating R-factors(gt) etc. and is not relevant to the choice of reflections for refinement. R-factors based on F^2^ are statistically about twice as large as those based on F, and R- factors based on ALL data will be even larger.

### Fractional atomic coordinates and isotropic or equivalent isotropic displacement parameters (Å^2^)

**Table:**

	*x*	*y*	*z*	*U*_iso_*/*U*_eq_	
O1	−0.5092 (2)	0.92971 (5)	0.52984 (13)	0.0215 (3)	
O2	−0.2912 (2)	0.83869 (5)	0.60229 (13)	0.0215 (3)	
O3	0.6369 (2)	0.91915 (5)	0.88880 (13)	0.0209 (3)	
N1	−0.0747 (2)	0.99667 (6)	0.64651 (14)	0.0164 (3)	
H1	−0.2351	1.0154	0.5860	0.020*	
C1	−0.0569 (3)	0.93435 (7)	0.66638 (17)	0.0167 (3)	
C2	0.1785 (3)	0.90548 (7)	0.74844 (17)	0.0179 (3)	
H2A	0.1879	0.8620	0.7624	0.022*	
C3	0.4006 (3)	0.94219 (7)	0.80976 (17)	0.0172 (3)	
C4	0.3838 (3)	1.00878 (7)	0.78808 (17)	0.0165 (3)	
C5	0.6021 (3)	1.04912 (7)	0.84392 (18)	0.0196 (3)	
H5A	0.7657	1.0325	0.8995	0.024*	
C6	0.5718 (3)	1.11260 (8)	0.81587 (18)	0.0215 (4)	
H6A	0.7157	1.1388	0.8513	0.026*	
C7	0.3247 (3)	1.13855 (7)	0.73390 (18)	0.0213 (3)	
H7A	0.3077	1.1818	0.7171	0.026*	
C8	0.1083 (3)	1.10119 (7)	0.67844 (17)	0.0192 (3)	
H8A	−0.0542	1.1187	0.6248	0.023*	
C9	0.1385 (3)	1.03559 (7)	0.70470 (17)	0.0163 (3)	
C10	−0.3094 (3)	0.89756 (7)	0.59208 (17)	0.0165 (3)	
C11	0.6630 (3)	0.85142 (7)	0.91289 (19)	0.0229 (4)	
H11A	0.8409	0.8411	0.9726	0.034*	
H11B	0.6036	0.8302	0.8198	0.034*	
H11C	0.5617	0.8382	0.9622	0.034*	
O1W	0.0737 (2)	0.75591 (5)	0.16811 (14)	0.0248 (3)	
H2	0.1349	0.7589	0.2622	0.037*	
H3	−0.0434	0.7242	0.1451	0.037*	
O2W	0.2854 (2)	0.76156 (6)	0.47021 (14)	0.0283 (3)	
H4	0.4092	0.7895	0.5074	0.042*	
H5	0.2167	0.7580	0.5317	0.042*	

### Atomic displacement parameters (Å^2^)

**Table:**

	*U*^11^	*U*^22^	*U*^33^	*U*^12^	*U*^13^	*U*^23^
O1	0.0150 (5)	0.0235 (5)	0.0221 (7)	0.0015 (4)	0.0045 (4)	0.0012 (4)
O2	0.0189 (5)	0.0199 (5)	0.0235 (6)	−0.0012 (4)	0.0073 (5)	0.0017 (4)
O3	0.0162 (5)	0.0211 (5)	0.0204 (6)	0.0024 (4)	0.0034 (4)	0.0024 (4)
N1	0.0153 (6)	0.0185 (6)	0.0135 (7)	0.0000 (5)	0.0046 (5)	−0.0006 (5)
C1	0.0171 (7)	0.0205 (7)	0.0137 (8)	−0.0010 (6)	0.0077 (6)	−0.0016 (6)
C2	0.0172 (7)	0.0189 (7)	0.0168 (8)	0.0004 (6)	0.0066 (6)	−0.0002 (6)
C3	0.0152 (7)	0.0244 (7)	0.0118 (8)	0.0026 (6)	0.0056 (6)	0.0004 (6)
C4	0.0154 (7)	0.0216 (7)	0.0126 (7)	0.0001 (6)	0.0061 (5)	−0.0011 (6)
C5	0.0160 (7)	0.0253 (7)	0.0153 (8)	−0.0016 (6)	0.0050 (6)	−0.0024 (6)
C6	0.0201 (7)	0.0245 (7)	0.0196 (9)	−0.0049 (6)	0.0083 (6)	−0.0049 (6)
C7	0.0236 (8)	0.0191 (7)	0.0207 (9)	−0.0016 (6)	0.0094 (6)	−0.0019 (6)
C8	0.0199 (7)	0.0209 (7)	0.0168 (8)	0.0010 (6)	0.0080 (6)	0.0000 (6)
C9	0.0167 (7)	0.0199 (7)	0.0122 (7)	−0.0005 (6)	0.0064 (6)	−0.0011 (6)
C10	0.0159 (7)	0.0201 (7)	0.0134 (8)	−0.0008 (5)	0.0061 (6)	−0.0004 (6)
C11	0.0214 (8)	0.0216 (7)	0.0228 (9)	0.0042 (6)	0.0070 (6)	0.0047 (6)
O1W	0.0256 (6)	0.0228 (5)	0.0244 (7)	−0.0035 (5)	0.0093 (5)	0.0004 (5)
O2W	0.0256 (6)	0.0335 (6)	0.0255 (7)	−0.0098 (5)	0.0110 (5)	−0.0068 (5)

### Geometric parameters (Å, °)

**Table:**

O1—C10	1.2461 (18)	C5—H5A	0.9300
O2—C10	1.2528 (18)	C6—C7	1.409 (2)
O3—C3	1.3337 (18)	C6—H6A	0.9300
O3—C11	1.4530 (18)	C7—C8	1.373 (2)
N1—C1	1.3332 (19)	C7—H7A	0.9300
N1—C9	1.3800 (19)	C8—C9	1.412 (2)
N1—H1	0.9437	C8—H8A	0.9300
C1—C2	1.385 (2)	C11—H11A	0.9600
C1—C10	1.528 (2)	C11—H11B	0.9600
C2—C3	1.391 (2)	C11—H11C	0.9600
C2—H2A	0.9300	O1W—H2	0.8586
C3—C4	1.425 (2)	O1W—H3	0.9083
C4—C9	1.411 (2)	O2W—H4	0.8759
C4—C5	1.419 (2)	O2W—H5	0.8743
C5—C6	1.370 (2)		
			
C3—O3—C11	117.76 (12)	C7—C6—H6A	119.7
C1—N1—C9	122.17 (13)	C8—C7—C6	121.28 (14)
C1—N1—H1	120.2	C8—C7—H7A	119.4
C9—N1—H1	117.5	C6—C7—H7A	119.4
N1—C1—C2	121.24 (14)	C7—C8—C9	118.44 (14)
N1—C1—C10	115.95 (13)	C7—C8—H8A	120.8
C2—C1—C10	122.80 (13)	C9—C8—H8A	120.8
C1—C2—C3	119.30 (14)	N1—C9—C4	119.14 (13)
C1—C2—H2A	120.3	N1—C9—C8	119.69 (13)
C3—C2—H2A	120.3	C4—C9—C8	121.16 (14)
O3—C3—C2	124.18 (14)	O1—C10—O2	127.72 (14)
O3—C3—C4	115.95 (13)	O1—C10—C1	116.14 (13)
C2—C3—C4	119.86 (13)	O2—C10—C1	116.14 (13)
C9—C4—C5	118.55 (14)	O3—C11—H11A	109.5
C9—C4—C3	118.28 (13)	O3—C11—H11B	109.5
C5—C4—C3	123.16 (14)	H11A—C11—H11B	109.5
C6—C5—C4	119.94 (14)	O3—C11—H11C	109.5
C6—C5—H5A	120.0	H11A—C11—H11C	109.5
C4—C5—H5A	120.0	H11B—C11—H11C	109.5
C5—C6—C7	120.61 (15)	H2—O1W—H3	103.8
C5—C6—H6A	119.7	H4—O2W—H5	106.9
			
C9—N1—C1—C2	0.7 (2)	C5—C6—C7—C8	−0.8 (3)
C9—N1—C1—C10	−178.65 (13)	C6—C7—C8—C9	−0.3 (2)
N1—C1—C2—C3	−0.8 (2)	C1—N1—C9—C4	0.0 (2)
C10—C1—C2—C3	178.50 (15)	C1—N1—C9—C8	179.18 (15)
C11—O3—C3—C2	−0.4 (2)	C5—C4—C9—N1	178.33 (14)
C11—O3—C3—C4	−179.67 (14)	C3—C4—C9—N1	−0.5 (2)
C1—C2—C3—O3	−179.06 (15)	C5—C4—C9—C8	−0.9 (2)
C1—C2—C3—C4	0.2 (2)	C3—C4—C9—C8	−179.68 (15)
O3—C3—C4—C9	179.73 (14)	C7—C8—C9—N1	−178.06 (15)
C2—C3—C4—C9	0.4 (2)	C7—C8—C9—C4	1.1 (2)
O3—C3—C4—C5	1.0 (2)	N1—C1—C10—O1	−5.3 (2)
C2—C3—C4—C5	−178.38 (15)	C2—C1—C10—O1	175.42 (15)
C9—C4—C5—C6	−0.3 (2)	N1—C1—C10—O2	175.44 (14)
C3—C4—C5—C6	178.51 (16)	C2—C1—C10—O2	−3.9 (2)
C4—C5—C6—C7	1.1 (3)		

### Hydrogen-bond geometry (Å, °)

**Table:**

*D*—H···*A*	*D*—H	H···*A*	*D*···*A*	*D*—H···*A*
N1—H1···O1	0.94	2.31	2.6638 (18)	102
N1—H1···O1^i^	0.94	1.84	2.7608 (18)	164
O1W—H2···O2W	0.86	1.89	2.7478 (19)	176
O1W—H3···O2^ii^	0.91	1.86	2.7685 (16)	177
O2W—H4···O2^iii^	0.88	1.88	2.7498 (18)	171
O2W—H5···O1W^iv^	0.87	1.91	2.7860 (19)	176
C6—H6A···O1W^v^	0.93	2.59	3.418 (2)	149
C8—H8A···O1^i^	0.93	2.53	3.229 (2)	132
C11—H11A···O1W^vi^	0.96	2.58	3.317 (2)	134
C11—H11B···O2^iii^	0.96	2.53	3.272 (2)	134

Symmetry codes: (i) −*x*−1, −*y*+2, −*z*+1; (ii) *x*, −*y*+3/2, *z*−1/2; (iii) *x*+1, *y*, *z*; (iv) *x*, −*y*+3/2, *z*+1/2; (v) −*x*+1, −*y*+2, −*z*+1; (vi) *x*+1, *y*, *z*+1.

## References

[bb1] Bernstein, J., Davis, R. E., Shimoni, L. & Chang, N.-L. (1995). *Angew. Chem. Int. Ed. Engl.* **34**, 1555–1573.

[bb2] Bruker (2009). *APEX2*, *SAINT* and *SADABS* Bruker AXS Inc., Madison, Wisconsin, USA.

[bb3] Campbell, S. F., Hardstone, J. D. & Palmer, M. J. (1988). *J. Med. Chem.* **31**, 1031–1035. 10.1021/jm00400a025 2896245

[bb4] Cosier, J. & Glazer, A. M. (1986). *J. Appl. Cryst.* **19**, 105–107.

[bb5] Elman, B., Högberg, S. A. G., Weber, M. & Muhammed, M. (1985). *Polyhedron*, **4**, 1197–1201.

[bb6] Loh, W.-S., Quah, C. K., Hemamalini, M. & Fun, H.-K. (2010*a*). *Acta Cryst.* E**66**, o2357. 10.1107/S1600536810032745 PMC3008075 21588699

[bb7] Loh, W.-S., Quah, C. K., Hemamalini, M. & Fun, H.-K. (2010*b*). *Acta Cryst.* E**66**, o2396. 10.1107/S1600536810033118 PMC3008022 21588729

[bb8] Markees, D. G., Dewey, V. C. & Kidder, G. W. (1970). *J. Med. Chem.* **13**, 324–326. 10.1021/jm00296a048 5418519

[bb9] Michael, J. P. (1997). *Nat. Prod. Rep.* **14**, 605–608.

[bb10] Morimoto, Y., Matsuda, F. & Shirahama, H. (1991). *Synlett*, **3**, 202–203.

[bb11] Reux, B., Nevalainen, T., Raitio, K. H. & Koskinen, A. M. P. (2009). *Bioorg. Med. Chem.* **17**, 4441–4447. 10.1016/j.bmc.2009.05.013 19477133

[bb12] Sasaki, K., Tsurumori, A. & Hirota, T. (1998). *J. Chem. Soc. Perkin Trans. 1*, pp. 3851–3856.

[bb13] Sheldrick, G. M. (2008). *Acta Cryst.* A**64**, 112–122. 10.1107/S0108767307043930 18156677

[bb14] Spek, A. L. (2009). *Acta Cryst.* D**65**, 148–155. 10.1107/S090744490804362X PMC2631630 19171970

[bb15] Zhou, P., O’Hagan, D., Mocek, U., Zeng, Z., Yuen, L.-D., Unkefer, C. J., Beale, J. M. & Floss, H. G. (1989). *J. Am. Chem. Soc.* **111**, 7274–7276.

